# Prognostic models for survival predictions in advanced cancer patients: a systematic review and meta-analysis

**DOI:** 10.1186/s12904-025-01696-4

**Published:** 2025-03-01

**Authors:** Mong Yung Fung, Yuen Lung Wong, Ka Man Cheung, King Hei Kelvin Bao, Winnie Wing Yan Sung

**Affiliations:** 1 Faculty of Medicine, The Chinese University of Hong Kong, Shatin, Hong Kong SAR, China; 2 Department of Clinical Oncology, Queen Elizabeth Hospital, Hong Kong SAR, China

**Keywords:** Prognostication, Prognostic model, Survival, Advanced cancer, Palliative care

## Abstract

**Background:**

Prognostication of survival among patients with advanced cancer is essential for palliative care (PC) planning. The implementation of a clinical point-of-care prognostic model may inform clinicians and facilitate decision-making. While early PC referral yields better clinical outcomes, actual referral time differs by clinical contexts and accessible. To summarize the various prognostic models that may cater to these needs, we conducted a systematic review and meta-analysis.

**Methods:**

A systematic literature search was conducted in Ovid Medline, Embase, CINAHL Ultimate, and Scopus to identify eligible studies focusing on incurable solid tumors, validation of prognostic models, and measurement of predictive performances. Model characteristics and performances were summarized in tables. Prediction model study Risk Of Bias Assessment Tool (PROBAST) was adopted for risk of bias assessment. Meta-analysis of individual models, where appropriate, was performed by pooling C-index.

**Results:**

35 studies covering 35 types of prognostic models were included. Palliative Prognostic Index (PPI), Palliative Prognostic Score (PaP), and Objective Prognostic Score (OPS) were most frequently identified models. The pooled C-statistic of PPI for 30-day survival prediction was 0.68 (95% CI: 0.62–0.73, n = 6). The pooled C-statistic of PaP for 30-day survival prediction was 0.76 (95% CI: 0.70–0.80, n = 11), while that for 21-day survival prediction was 0.80 (0.71–0.86, n = 4). The pooled C-statistic of OPS for 30-days survival prediction was 0.69 (95% CI: 0.65–0.72, n = 3). All included studies had high risk of bias.

**Conclusion:**

PaP appears to perform better but further validation and implementation studies were needed for confirmation.

**Supplementary Information:**

The online version contains supplementary material available at 10.1186/s12904-025-01696-4.

## Key message

This article describes a systematic review and meta-analysis of prognostic models for patients with advanced cancer and their performance statistics. The results indicate that Palliative Prognostic Score (PaP) has superior predictive accuracy but further validation and implementation studies were needed to confirm its value of clinical utility.

**Supplementary Information:**

The online version contains supplementary material available at 10.1186/s12904-025-01696-4.

## Introduction

In 2017, the American Society of Clinical Oncology (ASCO) updated its clinical practice guideline, highlighting the evidence base for early integration of palliative care alongside oncologic care [1]. The clinical benefits of palliative care integration include better quality of life, reduced depression, reduced hospital readmissions, improved satisfaction with care, and potential increase in survival [2, 3]. Moreover, referrals to palliative care services at earlier disease stages would result in greater improvements and overall fewer medical costs [4–8].

There is no universal consensus over the appropriate time for referrals. Data from randomized controlled trials suggest a minimal of 6 months for clinical benefits of palliative care to emerge [4, 5, 9–12]. ASCO guidelines recommend referrals to be made within 8 weeks of advanced cancer diagnosis [13]. However, such an early referral time may be unrealistic for certain healthcare systems. Referrals are also dependent on patients’ preferences. With the advent of personalized medicine, next-generation sequencing and targeted treatment, the disease trajectory is highly dependent on disease primary site, genotyping and availability of advanced treatments. Identifying the best timing to refer patients to palliative care service, particularly that which is not too late to enable full effect of holistic intervention, and not too early to lack relevance to service user, is therefore extremely challenging.

To facilitate decision-making, a reliable prediction of survival is needed. Multiple studies have suggested the inaccuracy of clinician prediction of survival in advanced cancer population [14, 15]. Patients with wrongly estimated survival may have poorer quality of life and higher symptom burden [14, 16]. While the prognosis of patients with advanced cancers amenable to effective life-prolonging treatment can be estimated using survival data derived from clinical trial reports, they were not immediately generalizable to cancer with no suitable novel treatments [17].

To address this gap, prognostic models have been developed for this particular group of patients, based on their clinical statuses or biologic factors. Many of these models are externally validated, but their implementation into clinical practice could be hindered by two main problems. First, some models involve non-routine biomarkers and complex calculations, rendering them unfeasible for everyday practice [18]. Second, studies on prognostic models differ in terms of patient characteristics, clinical settings, and methodologies. It is not straightforward for clinicians to extrapolate the data on model performance to their own clinical contexts [18].

We aim to make explicit the applicability of prognostic models for advanced cancer patients through two objectives. The primary objective of this study is to identify validated prognostic models and assess their performance. The secondary objective is to explore whether differences in patient characteristics and clinical settings across studies are associated with model performance.

## Methods

A systematic search was done on Medline, Embase, CINAHL Ultimate, and Scopus, with complementary reference mining from review articles. The search period was up to August 2022. Search terms were available in appendix I. Studies published in English full texts were eligible. Abstracts and conference articles were excluded. The study population should be targeted at patients with advanced cancer, defined as incurable, who were 18 or above. The study sample should consist of at least 2 cancer types, but not haematological malignancies. The prognostic models examined should include at least 2 factors and were validated internally or externally. The studies should report measures of model performance in terms of discrimination and/or calibration. Two reviewers independently screened out irrelevant articles based on title and abstract. Discrepancies were resolved by a third reviewer. Full texts were then retrieved for the remaining articles, for which detailed screening were done independently by the two reviewers. Disagreements were resolved by the same third reviewer.

Data extraction was done independently by two reviewers in accordance to CHARMS guidelines [19]. Risk of bias were assessed with PROBAST [20]. Where possible, missing data were obtained by contacting authors of original articles. Clinical heterogeneity was assessed in terms of clinical settings, patient characteristics, model types, and prediction timeframe.

Meta analysis was performed if adequate clinical homogeneity was established. Meta-analysis of the C-statistics with logit transformation was conducted using the packages METAFOR in R, to improve validity of its underlying assumptions. Restricted maximum likelihood (REML) estimation method was to calculate 95% confidence intervals for the average performance using the METAFOR package in R.

Test performance characteristics were summarized using a forest plot. Heterogeneity of prognostic model performance across studies was assessed by 95% prediction intervals (PI) and I^2^ statistic (I^2^). PI provides an estimated range within which the true effect size of a future study would be expected to fall 95% of the time. Wide PI suggest substantial heterogeneity in model performance. PI was calculated using the METAGEN package in R. I2 was estimated and that I2 > 50% was taken as signifying substantial heterogeneity.

Multilevel analysis and/or meta-regression were performed if more than 10 adequately homogeneous studies could be pooled.

The study was registered on PROSPERO with ID: CRD42023403263. The systematic search in database was carried out between 1st Aug 2022 to 31st Aug 2022. We included all studies publised on or before 31st Aug 2022.

## Results

**Fig. 1:**
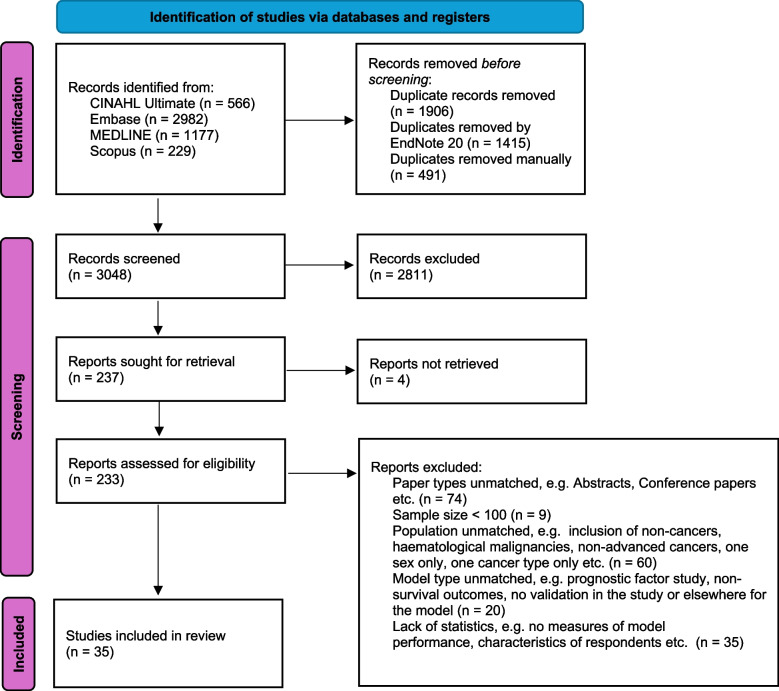
Flow Diagram for Study Selection

A total of 35 studies covering 35 types of prognostic models were included after the screening process detailed in Fig. 1. Characteristics of the included studies can be found in Table 1. 4 studies tested for survival in one week, 2 studies in one year, and the rest (*N* = 29) in between. 23 models utilize both clinical and objectively assessed parameters such as physical or laboratory measurements. 8 models adopted only clinical factors while 4 adopted only objective parameters.

**Table 1:** Characteristics of Included Studies

Study	Model	Sample size (Training set)	Sample Size (Validation set)	Validation Method	Clinical Setting	Treatment Status	Age	Measures of Survival	Follow-up Period	Overall Survival (95% CI, Days)
Chow et al., 2008 [21]	Survival Prediction Score (SPS) / Number of Risk Factors (NRF) (3-variable)	395	TV = 445, EV = 468	External, Temporal	Palliative RT clinic	Palliative RT	66 (Range: 24—91)	3-months, 6-months, 12-months	12 months	N/A
Chow et al., 2009 [22]	Survival Prediction Score (SPS) / Number of Risk Factors (NRF) (6-variable)	N/A	445	External	Palliative RT clinic	Palliative RT	69 (Range: 24–93)	3-months, 6-months, 12-months	12 months	19 weeks (range, 0.3–164 weeks)
Chiang et al., 2009 [23]	A proposed prognostic 7-day survival formula	374	353	Temporal	Palliative Care Unit (PCU)	N/A	67 (Inter-quartile range: 58—75)	7-days	N/A	N/A
Chow et al., 2009 [24]	Recrusive partitioning (2-variable)	395	TV = 445, EV = 468	External, Temporal	Palliative RT clinic	Palliative RT	66 (Range: 24—91)	3-months, 6-months, 12-months	12 months	20 weeks (Range: 0–116) for deceased, 52 weeks of median follow up for survivors
Scarpi et al., 2011 [25]	Palliative Prognostic Score (PaP) & Modified Palliative Prognostic Score—Delirium (D-PaP)	N/A	361	External	PCU	Palliative anti-neoplastic treatments	70	30-day survival	N/A	34
Cheng et al., 2012 [26]	Palliative Prognostic Index (PPI)	N/A	623	External	Hospice	Palliative anti-neoplastic treatments	62	1-week, 3-week, 6-week	until death	19 (15.3–23)
Durand et al., 2012 [27]	Cochin Risk Index Score (CRIS)	334	166	Internal	PCU	Not on any anti-neoplastic treatments	62 (Range: 18–93)	2-week	N/A	13 (+—9)
Maltoni et al., 2012 [28]	PaP, D-PaP, PPS, PPI	N/A	549	External	Hospice	N/A	71 (Range: 18–94)	21-days, 30-days	Until death or Jan, 31, 2011	22 (19-24)
Huang et al., 2014 [29]	Prognostic Scale for terminal hospitalized chinese cancer patients (8-variable)	181	128	Temporal	Cancer Center	N/A	64 (Range: 28—93)	30-days	Until death or 3 months after admission	20 (16.8—23.2)
Hung et al., 2014 [30]	PPI, change in PPI scores	N/A	1035	External	PCU	N/A	60.3	30-days, 60-days, 90-days	until death oe 3 months after admission	22 (20.3—23.7)
Kao et al., 2014 [31]	Initial PPI, change in PPI scores, combinatinon of initial PPI & change in PPI scores	1669	723	Temporal	PCU	N/A	58.7	30-days	until death oe 180 days after admission	32 (Range: 8—180)
Kim et al., 2014 [32]	PPS, PPI, PaP	N/A	415	External	Hospice	Palliative treatments (No longer on any anti-neoplastic treatments)	60.7 (Standard Deviation: +—12.6)	3-week and 4-week	until death	36.5 +—31.5
Yoon et al., 2014 [33]	Objective Prognostic score	N/A	104	External	Hospice, PCU	N/A	62.27 (Standard Deviation: +—12.86)	3-week	N/A	17 +—1.38
Chen et al., 2015 [34]	Objective Palliative Prognostic Score	234	N/A	Internal	Hospice	N/A	62.8 (Standard Deviation: +—13.6)	7-days	N/A	N/A
Chiang et al., 2015 [35]	A graphic tool to estimate individualized survival curves (5-variable)	286	N/A	Internal	Hospice	N/A	64 (Standard Deviation: +—13.6)	Individualized survival curve	Until death or end of study	18 (1–60)
R Mendis et al., 2015 [36]	PaP	N/A	644	External	PCU and home-based palliative services	N/A	PCU = 64.6 (Range 18.6 – 93.9) Home-based: 61.6 (Range 17.2 – 91.6)	30 days	N/A	N/A
Bourgeois etal., 2017 [37]	PRONOPALL score	N/A	262	External	PCU (Outpatient)	Palliative anti-neoplastic treatments	66 (Range: 37—88)	2-months and 6-months	N/A	186
Yoon et al., 2017 [38]	Objective Prognostic Score (OPS)	N/A	217	External	PCU	Not on anti-neoplastic treatments	65.1 (Standard Deviation: +—12.8)	3-week	N/A	14 (11.6—16.5)
Adelson et al., 2018 [39]	Imminent Mortality Predictor for Advanced Cancer (IMPAC)	468	200	Internal	N/A	N/A	63 (Standard Deviation: +—12)	90-day	N/A	47
C. Palomar-Munoz et al., 2018 [40]	PPI (On admission, on admision w/ concomitant diseases, on discharge)	N/A	332	External	PCU	Not on anti-neoplastic treatments	71 (Standard Deviation: +—13)	3 weeks, 6 weeks	death or study cllosure time	15 (IQR: 6–36)
Ermacora et al., 2018 [41]	PaP, OPS, PPI	N/A	334	External	PCU, hospice	Nogon anti-neoplastic treatments	72	30 days	N/A	14 (Range: 0–544)
Hamano et al., 2018 [42]	Objective Prognostic Index for Advanced cancer (OPI-AC), PiPS-B, PaP, SAP model	1039	N/A	Internal	PCU and home-based palliative services	N/A	67.7 (Standard Deviation: +—13.1)	7-days, 14-days, 30-days, 56-days, 90-days	Until death or 6 months after enrolment	33 (13–85)
Zhao et al., 2019 [43]	Prognostic nomogram for overall survival in mixed advanced cancer patients	247	131	Temporal	PCU	N/A	N/A	90 days	death or study closure	48 (Training), 52 (Validation)
Arkin et al., 2020 [44]	Artificial neural networks and logistic regression for 30-days survival prediction	133	28, 28	Internal	PCU (Outpatient)	Palliative anti-neoplastic treatments	64.5 (Standard Deviation: +—11.6)	30 days	death	N/A
Hum et al., 2020 [45]	PROgnostic model for Advanced Cancer (PRO-MAC)	650	280	Internal	PCU	N/A	73 (Standard Deviation: +—19.5)	30 days, 90 days	2 years	42
Miyagi et al., 2020 [46]	Prognositc model using routine laboratory results, PaP, PPI	N/A	225	External	PCU	Not on anti-neoplastic treatments	69.6 +—11.8	N/A	180 days or until death	30
Yang et al., 2021 [47]	Data mining techniques, e.g. random forest algorithms	310	N/A	Internal	Palliative home care service	Palliative anti-neoplastic treatments	N/A	Classification into < 30, 30–90, > 90	N/A	N/A
Chan et al., 2022 [48]	Rothman Index, Supportive and Palliative Care indicator tools	N/A	227	External	PCU	N/A	66 (Standard Deviation: +—9.7)	6 months	N/A	N/A
Hiratsuka et al., 2022_a [49]	PaP and PaP-CPS	N/A	Japan = 1422, Korea = 320, Taiwan = 330	External, Temporal	PCU	N/A	Japan = 74 (66—81)	30-days	6 months or until death	Japan = 19 (17.3–20.7), Korea = 23 (20.5–25.8), Taiwan = 15(12.6—17.5)
Hiratsuka et al., 2022_b [50]	Objective Prognostic Score vs Palliative Prognostic Score	N/A	Japan = 1360, Korea = 268	External	PCU	N/A	Japan = 72.7 +—12.2, Korea = 68.8 +—12.3	3-week, 30-day	6 months or until death	Japan = 18 (16.3–19.7), Korea = 22 (18.9—25)
Hiratsuka et al., 2022_c [51]	PPS, PPI, PaP, CPS	N/A	1896	External	PCU (Inpatient)	N/A	72.4 +—12.3	7, 14, 30, 60-days	6 months or until death	19 (2- 140.2)
Owusuaa et al., 2022 [52]	Surprise question, clinical model, extended model	867	N/A	Internal	PCU	Palliative anti-neoplastic treatments	66 (56—72)	1-year survival	maximum 1 year	N/A
Preto et al., 2022 [53]	modified Barretos Prognostic nomogram (with / without laboratory values)	215	276	Temporal	PCU (Outpatient)	67.8% on Palliative anti-neoplastic treatments	60.2	30, 90, 180 days	N/A	124 (104.2—143.7)
Scarpi et al., 2022 [54]	PaP (Nomogram)	519	IV = 451, EV = 549	Internal, External	Hospice	Palliaitve hormonal treatment or RT, other antineoplastic therapy excluded	71 (range: 18–94)	15, 30, 60 days	N/A	59 (52—72)
Zachariah et al., 2022 [55]	Machine learning (Gradient-boosted trees binary classifier) vs clinical prediction	2041	N/A	Temporal	Cancer center	N/A	62.6 (range:18–96)	3-months	N/A	N/A

Fields marked as N/A indicate that the information was not reported in the original study.

Prognostic Models; *CRIS* Cochin Risk Index Score, *D-PaP* Modified Palliative Prognostic Score – Delirium, *IMPAC* Imminent Mortality Predictor for Advanced Cancer, *NRF* Number of Risk Factors, *OPS* Objective Prognostic Score, *PaP* Palliative Prognostic Score, *PiPS* Prognosis in Palliative Care Study, *PPI* Palliative Prognostic Index, *PRO-MAC* PROgnostic model for Advanced Cancer, *SAP* Six Adaptable Prognosis Prediction Model, *SPS* Survival Prediction Score, Study Characteristics; *EV* External Validation, *TV* Temporal Validation, *PCU* Palliative Care Unit. *RT* Radiotherapy

Out of the 8 clinical-only models, 15 prognostic factors were identified with performance status like ECOG, KPS, etc. being the most included factor (*n* = 8). Other commonly included factors include distant metastases (*n* = 4), edema (*n* = 4) and poor oral intake (*n* = 4).

Out of the 4 objective models, 9 prognostic factors were identified with hypoalbuminemia being the most included prognostic factor (*n* = 4). Other commonly included prognostic factors include heart rate (*n* = 3) and urea (*n* = 3).

Out of the 23 mixed models, 46 prognostic factors were identified with performance status being the most included prognostic factor (*n* = 19), followed by WBC count (*n* = 11), metastases (*n* = 10), dyspnea (*n* = 8) and poor oral intake (*n* = 7).

Table 2 lists the factors involved in each model and their nature. Most studies contain a mixture of clinical and biological factors.

**Table 2:** Factors of Prognostic Models

Models	Objective Factors		Clinical Factors	
	Continuous	Categorical	Continuous	Categorical
Palliative Prognostic Index (PPI)				**✓**
Combination of initial palliative prognostic Index (PPI) and week 1 PPI				**✓**
PPI on discharge / PPI on admission for patients with acute concomitant disease				**✓**
Survival Prediction Score (SPS): 3-variable model				**✓**
Number of risk factors (NRF): 3-variable model				**✓**
A proposed prognostic 7-day survival formula	**✓**			**✓**
Recursive partitioning: 2-variable model				**✓**
Survival Prediction Score (SPS): 6-variable model				**✓**
Number of risk factors (NRF): 6-variable model				**✓**
Palliative Prognostic Score (PaP)	**✓**			**✓**
Modified Palliative Prognostic Score—Delirium (D-PaP)	**✓**			**✓**
Palliative Prognostic Score—Nomogram (PaP-Nomogram)	**✓**			**✓**
Cochin Risk Index Score (CRIS)		**✓**		**✓**
Palliative Performance Scale (PPS)				**✓**
Prognostic Scale for terminal hospitalized chinese cancer patients (8-variable)				**✓**
A graphic tool to estimate individualized survival curves (5-variable)				
PRONOPALL score (4-variables)				**✓**
Objective Prognostic Score (OPS)		**✓**		**✓**
Imminent Mortality Predictor for Advanced Cancer (IMPAC)	**✓**	**✓**	**✓**	**✓**
Objective Prognostic Index for advanced cancer (OPI-AC) (7-days)	**✓**			
Objective Prognostic Index for advanced cancer (OPI-AC) (14-days)	**✓**			
Objective Prognostic Index for advanced cancer (OPI-AC) (30-days)	**✓**			
Prognosis in Palliative Care study (PiPS-B14/56)				**✓**
Six adaptable prognosis prediction (SAP) model	**✓**			
Nomogram based parameters to predict 90-days survival		**✓**		**✓**
Artificial Neural network for 30-days survival prediction	**✓**		**✓**	**✓**
Logistic regression for 30-days survival	**✓**		**✓**	**✓**
Prognostic model for advanced cancer (PRO-MAC)	**✓**			**✓**
Modified Barretos Prognostic Nomogram (BPN)—with laboratory values	**✓**	**✓**		**✓**
Modified Barretos Prognostic Nomogram (BPN)—without laboratory values		**✓**		**✓**
Machine learning (Gradient-boosted trees binary classifier)	**✓**	**✓**	**✓**	**✓**
Objective Palliative Prognostic Score		**✓**		**✓**
Clinical Model		**✓**		**✓**
Extended Model		**✓**		**✓**
Rothman Index		**✓**		**✓**
Supportive and Palliative Care Indicators Tool		**✓**		**✓**
Data mining techniques (random forest algorithms, support-vector machine algorithms, back-propagation neural network algorithms)	**✓**	**✓**	**✓**	**✓**

1. Please refer to the appendix for the full list of variables of included studies

2. Blank fields indicate that these variables were not utilized in the models

*KPS* Karnofsky Performance Status, *ECOG* Eastern Cooperative Oncology Group (ECOG) Performance Status,

*ESAS* Edmonton Symptom Assessment System , *AED* Accident & Emergency Department, *TNM* Tumor, Node, Metastasis, *PPS* Palliative Performance Scale

Table 3 captures performance of models demonstrated in each study. C-indices presented in the included studies ranged from 0.61–0.86. Palliative Prognostic Index (PPI) and Palliative Prognostic Score (PaP) were the most extensively validated models, followed by Objective Prognostic Score (OPS) and Palliative Performance Score (PPS). Details of classification, discrimination, and calibration statistics can be found in Table 3 Characteristics of Included Studies

**Table 3:** Performance Statistics of Models

Model	Author (Year)	Duration	Cut-off values	Sen (%) (95% CI)	Spe (%) (95% CI)	PPV(%) (95% CI)	NPV(%) (95% CI)	C-index (95% CI)
**Palliative Prognostic Index (PPI)**	Cheng et al., 2012 [26]	3 weeks^a^	6	71	68	81	56	0.68
	Maltoni et al., 2012 [28]	30 days^a^	6	73.7 (68.4–79)	67.1 (61.7–72.6)	67.8 (62.4–73.2)	73.1 (67.7–78.5)	0.62 (0.60–0.65)
	Hung, 2014 [30]	30 days^a^	8	58.9	64.8	73.7	48.4	0.66 (0.63—0.69)
	Kao et al., 2014 [31]	30 days	5	79.5	50.8	57.1	75	0.63 (0.61–0.65)
	Kim et al., 2014 [32]	3 weeks	5	60	63.3	45.4	75.7	0.65 (0.61—0.70)
	C. Palomar-Munoz et al., 2018 [40]	3 weeks^a^	6	79	51	66	66	N/A
	Ermacora et al., 2018 [41]	30 days	N/A	N/A				0.72 (0.67–0.77)
	Miyagi et al., 2020 [46]	3 weeks	N/A	N/A				0.76 (0.64–0.88)
	Hiratsuka et al., 2022_c [51]	30 days^a^	N/A	N/A				0.74 (0.72—0.76)
**Combination of initial palliative prognostic Index (PPI) and week 1 PPI**	Kao et al., 2014 [31]	30 days	4	66.9	77	70.6	73.8	0.71 (0.69—0.73)
**Survival Prediction Score (SPS): 3-variable model**	Chow et al., 2008 [21]	N/A	N/A	N/A				0.63
**Number of risk factors (NRF): 3-variable model**	Chow et al., 2008 [21]	N/A	N/A	N/A				0.63
**A proposed prognostic 7-day survival formula**	Chiang et al., 2009 [23]	1 week	0.2	71	75.7	26.8	90.1	N/A
**Recursive partitioning: 2-variable model**	Chow et al., 2009 [24]	N/A	N/A	N/A				0.61
**Survival Prediction Score (SPS): 6-variable model**	Chow et al., 2009 [22]	N/A	N/A	N/A				0.65
**Number of risk factors (NRF): 6-variable model**	Chow et al., 2009 [22]	N/A	N/A	N/A				0.65
**Palliative Prognostic Score (PaP)**	Scarpi et al., 2011 [25]	30 days	N/A					N/A
	Maltoni et al., 2012 [28]	30 days^a^	9	69.9 (64.4–75.4)	83.7 (79.3–88.2)	80.2 (75.0–85.3)	74.8 (70.0–79.5)	0.72 (0.70–0.73)
	Kim et al., 2014 [32]	3 weels	10	72.9	74.2	59	84.3	0.81 (0.77—0.85)
	[42]	30-days	N/A	N/A				0.87 (0.85—0.89)
	Ermacora et al., 2018 [41]	30 days	N/A	N/A				0.82 (0.77–0.86)
	Miyagi et al., 2020 [46]	3 weeks	N/A	N/A				0.86 (0.79–0.93)
	Hiratsuka et al., 2022_a [49]	30 days	N/A	N/A				Japan = 0.75 (0.73–0.78), Korea = 0.66 (0.6—0.72), Taiwan = 0.67 (0.61—0.74)
	Hiratsuka et al., 2022_b [50]	30 days^a^	N/A	91.1 (88.9–92.9)	40.2 (36.1–44.4)	68.8 (67.3–70.4)	75.6 (70.8–79.8)	Japan = 0.70 (0.68—0.73) Korea = 0.71 (0.64—0.77)
	Hiratsuka et al., 2022_c [51]	30 days^a^	N/A	N/A				0.84 (0.82—0.86)
	R. Mendis et al., 2015 [36]	30 days	N/A	N/A				0.71 (0.68–0.74)
**Modified Palliative Prognostic Score—Delirium (D-PaP)**	Hamano et al., 2018 [25]	30 days	N/A	N/A				N/A
	Maltoni et al., 2012 [28]	3 weeks	9	72.9 (67.6–78.3)	80.2 (75.6–84.9)	77.6 (72.4–82.8)	75.9 (71.1–80.8)	76.7 (72.7—80.7)
**Palliative Prognostic Score—Nomogram (PaP-Nomogram)**	Scarpi et al., 2022 [54]	15-days^a^	Various survival probability based on nomogram points	N/A				0.74 (0.72—0.75)
**Cochin Risk Index Score (CRIS)**	Durand et al., 2012 [27]	2 week	7	70	62	78		N/A
**Palliative Performance Scale (PPS)**	Maltoni et al., 2012 [28]	3 weeks^a^	60	N/A				0.63 (0.60–0.66)
	Kim et al., 2014 [32]	3 weeks ^a^	30	65	69.8	52.3	79.7	0.729 (0.68—0.77)
	Hiratsuka et al., 2022_c [51]	30 days ^a^	Not specified	N/A				0.73 (0.70—0.75)
**Prognostic Scale for terminal hospitalized chinese cancer patients (8-variable)**	Huang et al., 2014 [29]	30 days	4	70	77	78	73	N/A
**A graphic tool to estimate individualized survival curves (5-variable)**	Chiang et al., 2015 [35]	Analysis by survival curve only	N/A	N/A				0.69
**PRONOPALL score (4-variables)**	Bourgeois etal., 2017 [38]	2 months^a^	N/A	89.4	60.9	41.2	76.9	0.81 (0.75—0.87)
**Objective Prognostic Score (OPS)**	Yoon et al., 2014 [33]	3 week	3	83.6	56.8	77.8	65.6	0.74
	Yoon et al., 2017 [38]	3-week	3	73.6	66.2	79.8	58	0.74 (0.68—0.81)
	Ermacora et al., 2018 [41]	30 days	N/A	N/A				0.70 (0.64–0.75)
	Hiratsuka et al., 2022_b [50]	30 days^a^	3	43.6 (40.1–47.1)	87.8 (84.7–90.4)	83.8 (80.3–86.7)	51.7 (50.0–53.5	Japan: 0.70 (0.68—0.73) Korea: 0.71 (0.64—0.77)
**Imminent Mortality Predictor for Advanced Cancer (IMPAC)**	Adelson et al., 2018 [39]	90-days^a^	50%	40	N/A	60	N/A	0.72
**Objective Prognostic Index for advanced cancer (OPI-AC) (7-days)**	Hamano et al., 2018 [42]	7-days ^a^	N/A	N/A				0.77 (0.66—0.87)
**Prognosis in Palliative Care study (PiPS-B14/56)**	Hamano et al., 2018 [42]	14-days ^a^	N/A	N/A				0.86 (0.84—0.89)
**Six adaptable prognosis prediction (SAP) model**	Hamano et al., 2018[42]	30-days ^a^	N/A	N/A				0.74 (0.65—0.83)
**Nomogram based parameters to predict 90-days survival**	Zhao et al., 2019 [43]	90 days	N/A	N/A				0.75 (0.70—0.80)
**Artifical Neural network for 30-days survival prediction**	Arkin et al., 2020 [44]	30-days	N/A	38	100	N/A	N/A	0.86
**Logistic regression for 30-days survival**	Arkin et al., 2020 [44]	30-days	N/A	48	84	N/A	N/A	0.76
**Prognostic model for advanced cancer (PRO-MAC)**	Hum et al., 2020 [45]	30-days ^a^	4	66.9	68.1	57.1	76.5	0.73 (0.69–0.75)
**Supportive and Palliative Care indicator tools**	Chan et al., 2022 [48]	6 months	N/A	83.5	61	66.4	80	N/A
**Rothman Index**	Chan et al., 2022 [48]	6 months	60	69.7	11.9	42.2	29.8	N/A
**Patient-Generated Subjective Global Assessment Short form (PG-SGA SF)**	Cunha et al., 2022	90 days	15	60.2	70.1	N/A	N/A	0.75 (0.67—0.80)
**Modified Barretos Prognostic Nomogram (BPN)—with laboratory values**	Preto et al., 2022 [53]	30-days^a^	N/A	N/A				0.78 (0.74—0.81)
**Modified Barretos Prognostic Nomogram (BPN)—without laboratory values**	Preto et al., 2022 [53]	30-days^a^	N/A	N/A				0.74 (0.71—0.77)
**Machine learning (Gradient-boosted trees binary classifier)**	Zachariah et al., 2022 [55]	90-days	N/A	29.5	N/A	60	N/A	0.81 (0.83—0.91)
**Objective Palliative Prognostic Score**	Chen et al., 2015 [34]	1 week	3 out of 6 variables reached	68.8	86	55.9	91.4	0.82 (0.75—0.89)
**Clinical Model**	Owusuaa et al., 2022 [52]	1-year	40%	80	69	65	83	0.76 (0.73–0.78)
**Extended Model**	Owusuaa et al., 2022 [52]	1-year	40%	76	72	66	81	0.78 (0.76–0.80)
**Data mining techniques (random forest algorithms, support-vector machine algorithms, back-propagation neural network algorithms)**	Yang et al., 2021 [47]^b^	Classification into < 30 days, 30–90 and > 90 days	N/A	N/A				N/A

Fields marked as N/A indicate that the information was not reported in the original study.

^a^Authors also evaluated model performance at prediction intervals other than those listed in this table

^b^The author reported model accuracy rather than classification statistics and/or C-statistics:

random forest algorithm: 81.94% (SD: +- 6.12%), back-propagation neural network: 72.90% (SD: +- 8.08%)

*Sen* sensitivity, *Spe* specificity, *PPV* positive predictive value, *NPV* negative predictive value

**Fig. 2:**
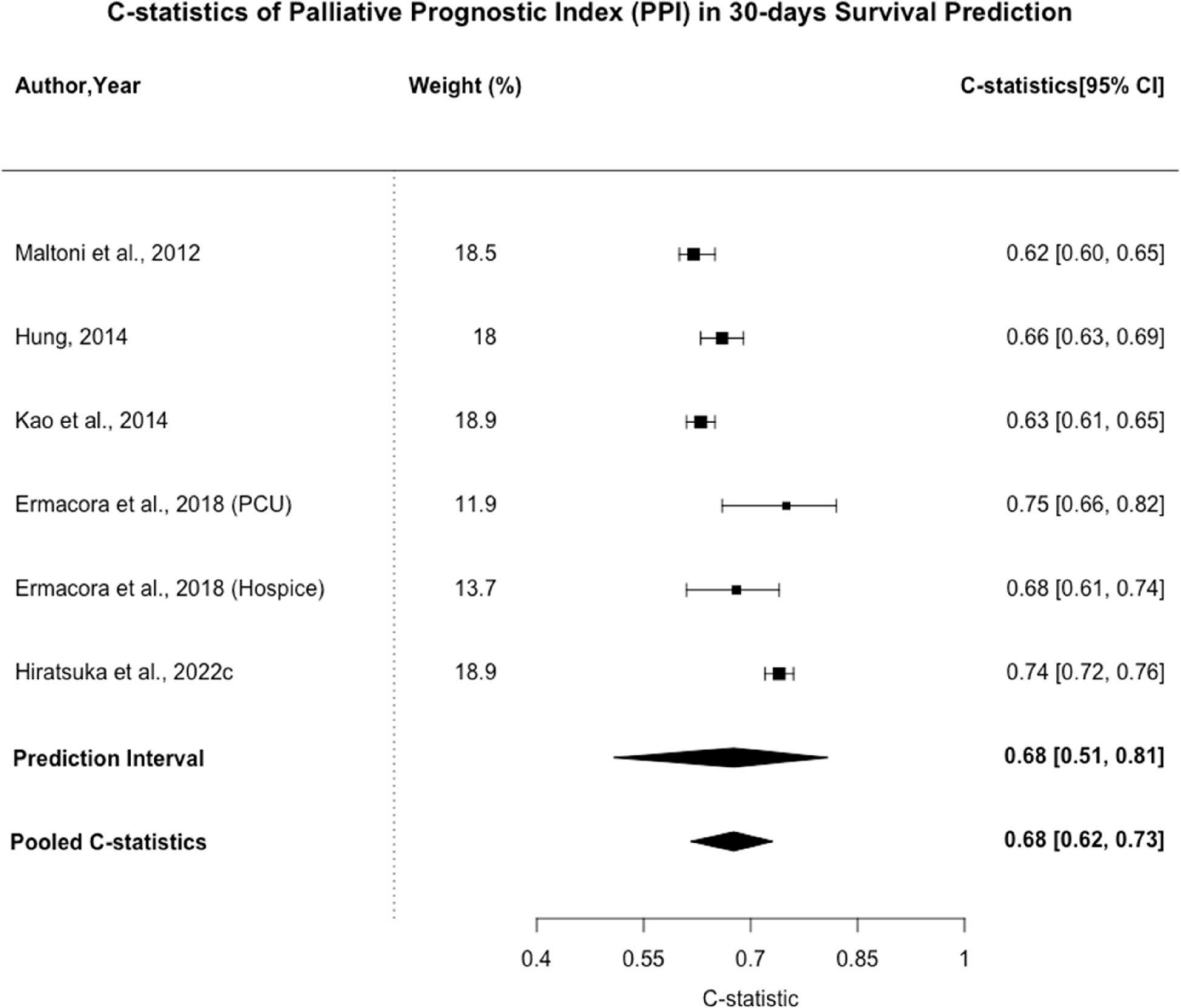
C-statistics of Palliative Prognostic Index (PPI) in 30-days Survival Prediction

Palliative Prognostic Index (PPI) was validated in 9 of the included studies. It consists of 5 clinical factors, namely palliative performance scale, oral intake, edema, dyspnea, and delirium. The pooled C-statistic for 30-day survival prediction was 0.68 (95% CI: 0.62–0.73, n = 6) as shown in Fig. 2. The 95% Prediction Interval (PI) was [0.51–0.81]. The I^2^ statistic was 93.9% (95% CI: 89.4%—96.5%), indicating significant heterogeneity. While PPI was typically compiled at initial assessment in palliative care service, Kao et al. investigated the prognostic value of combining both initial and change in PPI score. The C-statistic for predicting 30-day survival was shown to be significantly higher with the combined initial PPI and ∆score (C-statistic, 0.71; 95% confidence interval (CI), 0.694–0.731) than with the initial PPI (C-statistic, 0.63; 95% CI, 0.61–0.65), week 1 PPI (C-statistic, 0.67; 95% CI, 0.652–0.690), or ∆score (C-statistic, 0.64; 95% CI, 0.62–0.66) alone. [31].

**Fig. 3:**
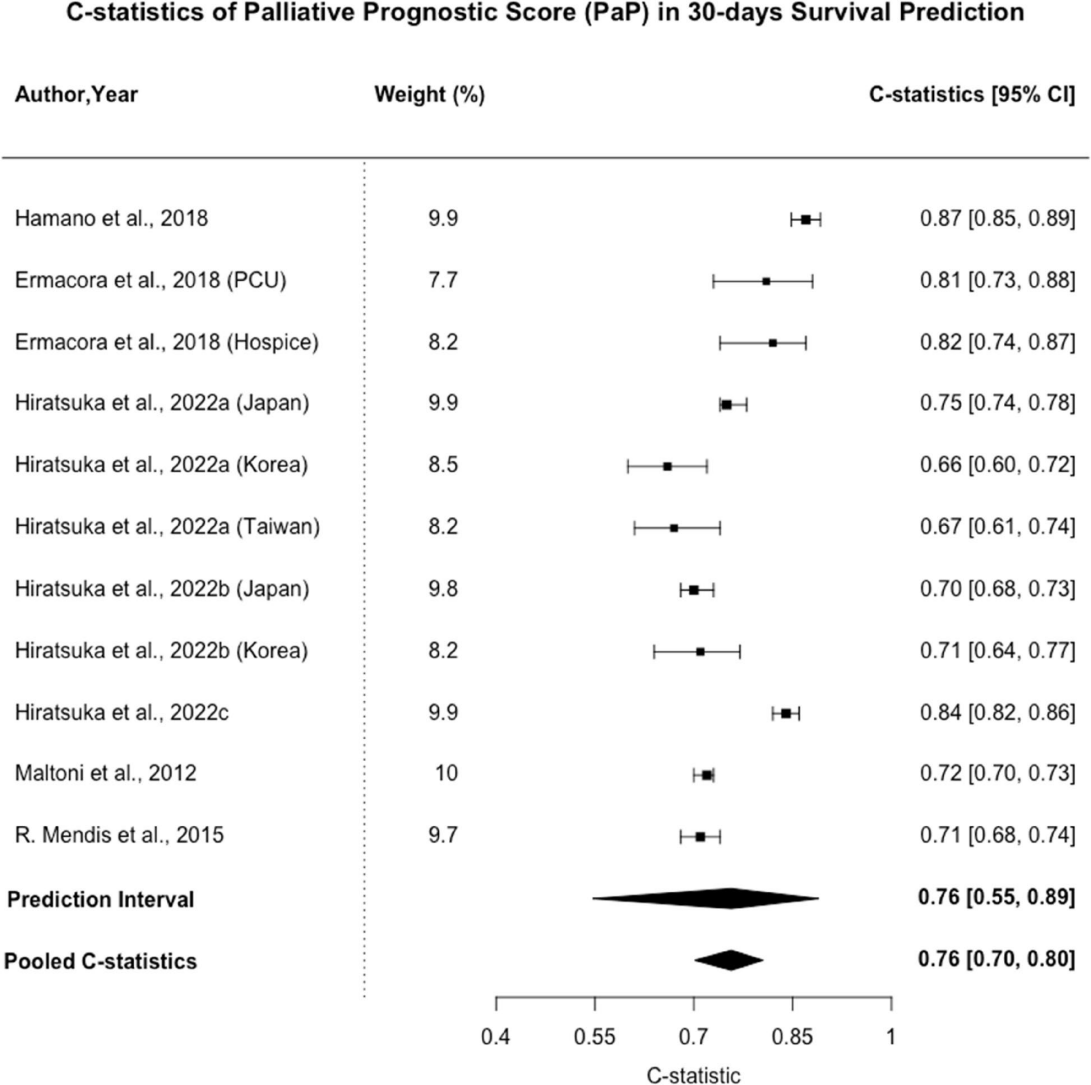
C-statistics of Palliative Prognostic Score (PaP) in 30-days Survival Prediction

**Fig. 4:**
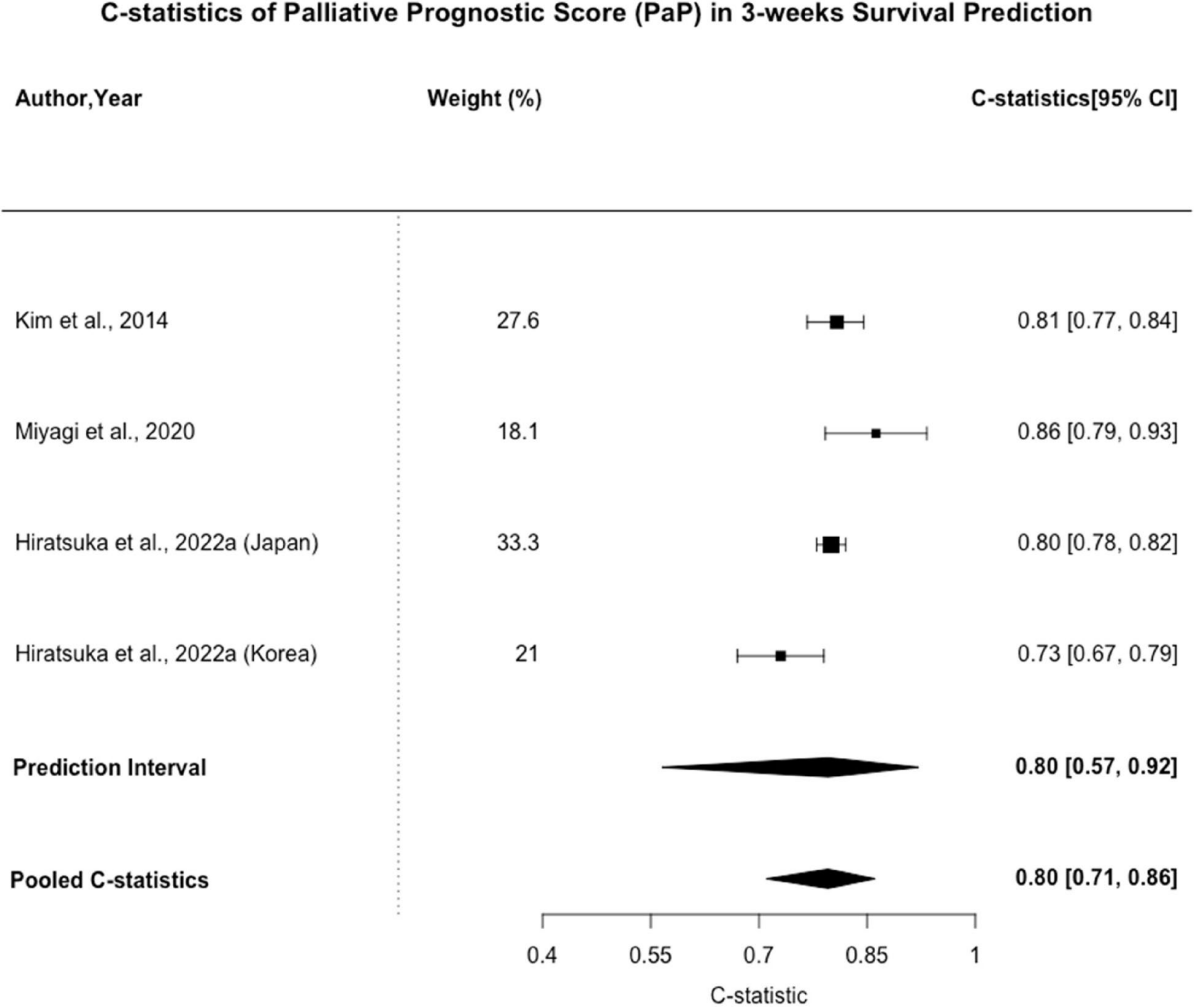
C-statistics of Palliative Prognostic Score (PaP) in 3-weeks Survival Prediction

Palliative Prognostic Score (PaP) was validated in 10 of the included studies. It consists of 4 clinical factors, including dyspnea, anorexia, KPS, and clinical prediction of survival, as well as 2 laboratory factors, including white cell count and lymphocyte percentage. As shown in Figs. 3, 4, the pooled C-statistic for 30-day survival prediction was 0.76 (95% CI: 0.70–0.80, *n* = 11), while that for 21-day survival prediction was 0.80 (0.71–0.86, *n* = 4). The 95% PI for 30-day and 21-day survival predictions were [0.54 – 0.90] and [0.57 – 0.92] respectively. The I^2^ statistics were 95.9% (95% CI: 94.2%—97.1%) and 64.3% (95% CI: 0 – 87.9%) for 30-day and 21-day survival predictions, respectively. Two additional studies assessed the incorporation of delirium to the PaP model (D-PaP). Scarpi et al. presented a marginally higher K statistics for 30-day survival with D-PaP (0.860, 95% CI: 0.817– 0.880) than PaP (0.853, 95% CI: 0.823–0.877) for the PaP score [25]. Maltoni et al. found that D-PaP had a C-statistic of 0.73 (95% CI: 0.71–0.74) compared to PaP which had a C-statistic of 0.72 (95% CI: 0.70–0.73) [28]. We emphasize that due to a relatively small number of studies included in the meta-analysis, the prediction intervals and I^2^ value presented should not be used to draw strong conclusions about heterogeneity or the range of true effects.

**Fig. 5:**
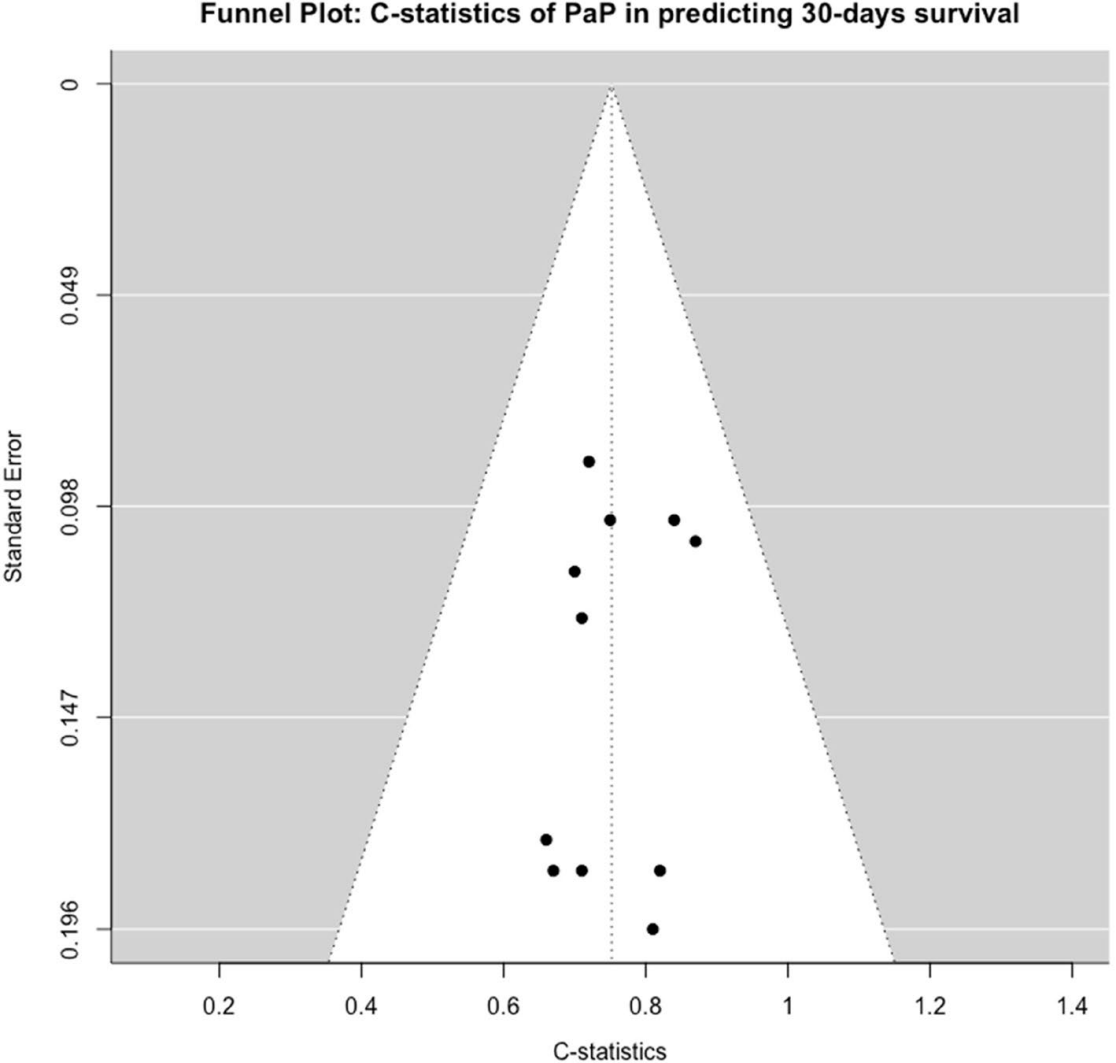
Funnel Plot: C-statistics of PaP in predicting 30-days survival

As shown in Fig. 5, the funnel plot of C-statistics for PaP showed mild asymmetry, with a slight clustering of studies towards higher C-statistics, potentially suggesting a mild publication bias. No extreme outliers were observed.

Objective Prognostic Score (OPS) was validated in 5 of the included studies. It consists of 3 clinical factors, namely Eastern Cooperative Oncology Group (ECOG) Performance Status, dyspnea at rest, and oral intake, along with 3 laboratory factors, namely white cell count, serum bilirubin, and serum creatinine. The C-statistic of OPS for 30-days survival prediction ranged from 0.68 (95% CI: 0.58 – 0.77) [41] to 0.71 (95% CI: 0.64 – 0.77) [50].

**Fig. 6:**
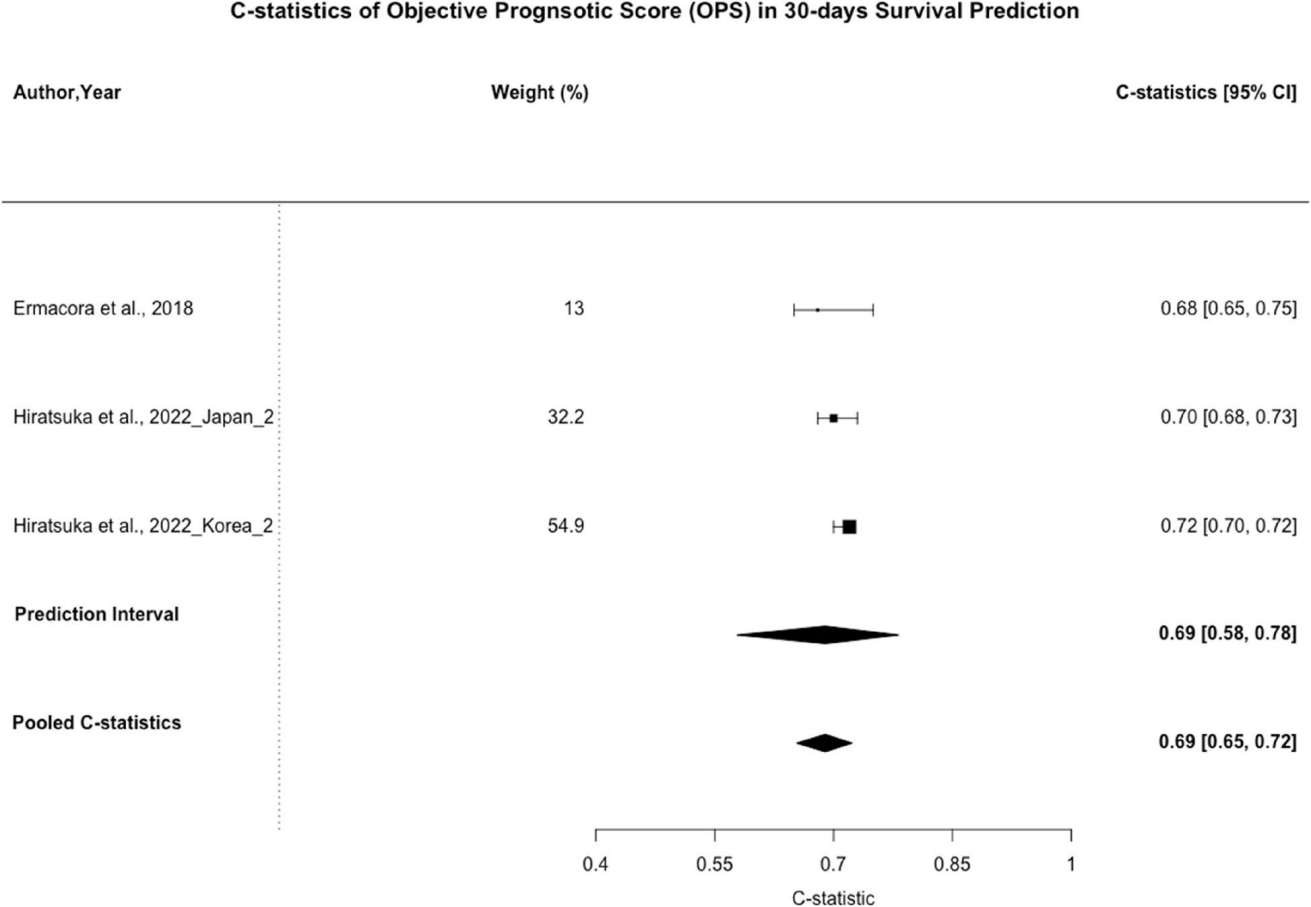
C-statistics of Objective Prognostic Score (OPS) in 30-days Survival Prediction

As shown in Fig. 6, the pooled C-statistic for 30-day survival prediction was 0.69 (95% CI: 0.65–0.72, n = 3). The 95% PI for 30-day survival predictions was [0.58 – 0.78]. The I^2^ statistics was 92.1% (95% CI: 87.9%—98.4%) for 30-day survival prediction.

Palliative Performance Score (PPS) was validated in 3 of the included studies. It comprises 5 clinical factors, including ambulation, activity level, self-care, intake, and level of consciousness. Meta-analysis on C-statistics of PPS was not performed due to insufficient number of studies with adequate clinical homogeneity.

Multilevel meta-analysis including 18 datasets from 14 unique studies was conducted to compare the performance of PaP and PPI in predicting 30-days survival, accounting for within-study dependence. The difference in the C-statistic between PaP and PPI was statistically significant (p < 0.0001). The pooled C-statistic of PaP was 0.76 (95% CI: 0.70 – 0.80). In contrast, the C-statistic of PPI was estimated to be 0.0485 (95% CI: 0.0388 – 0.0583) lower than that of PaP. The variance component analysis indicated minimal variability between datasets (σ2 = 0.0046). The nested Study/Model effect showed negligible variance, suggesting that the relative performance of PaP and PPI was consistent across studies. However, these findings should be interpreted with caution due to the relatively small number of studies included.

**Fig. 7:**
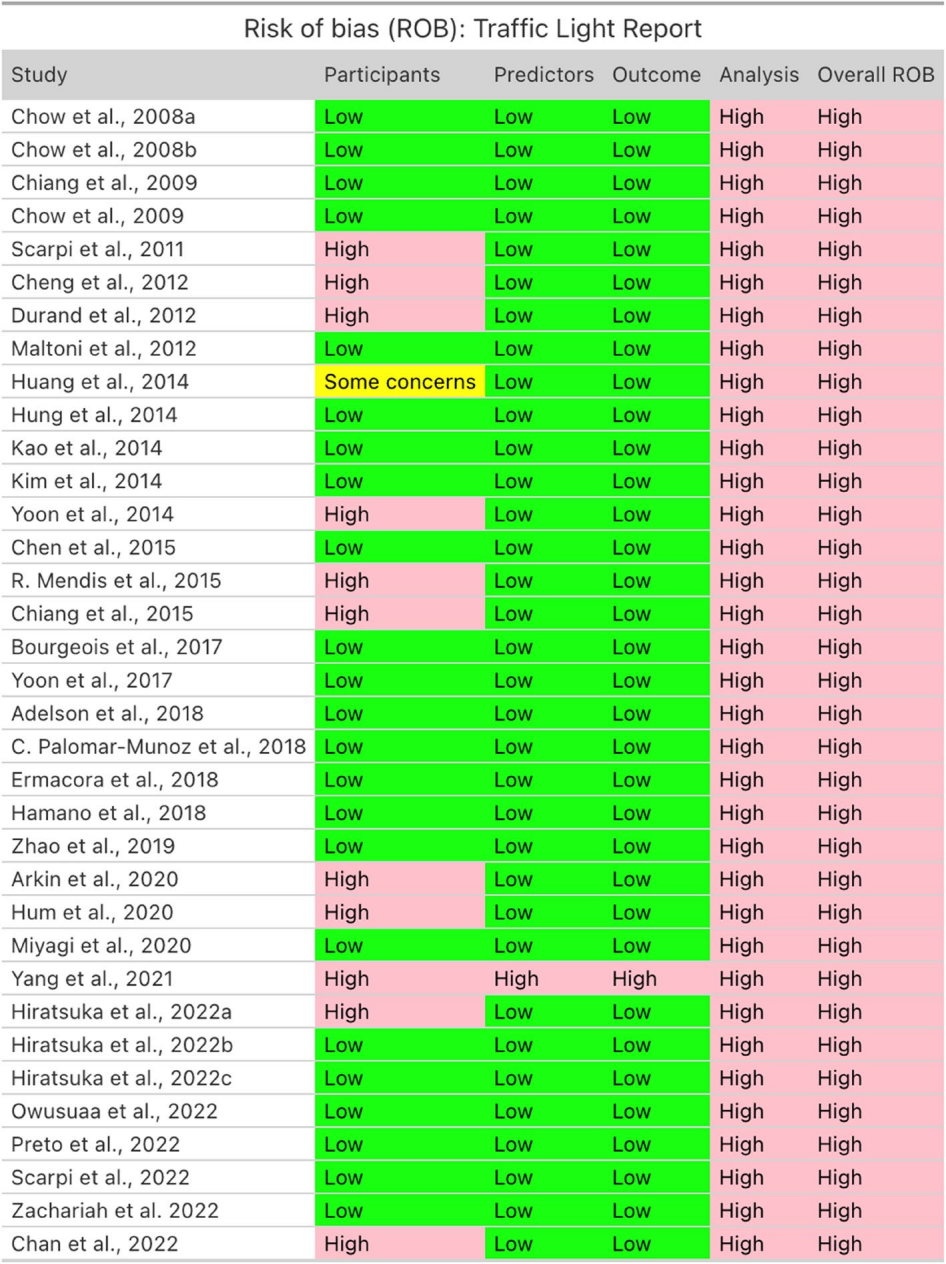
Risk of bias (ROB): Traffic Light Report

**Fig. 8:**
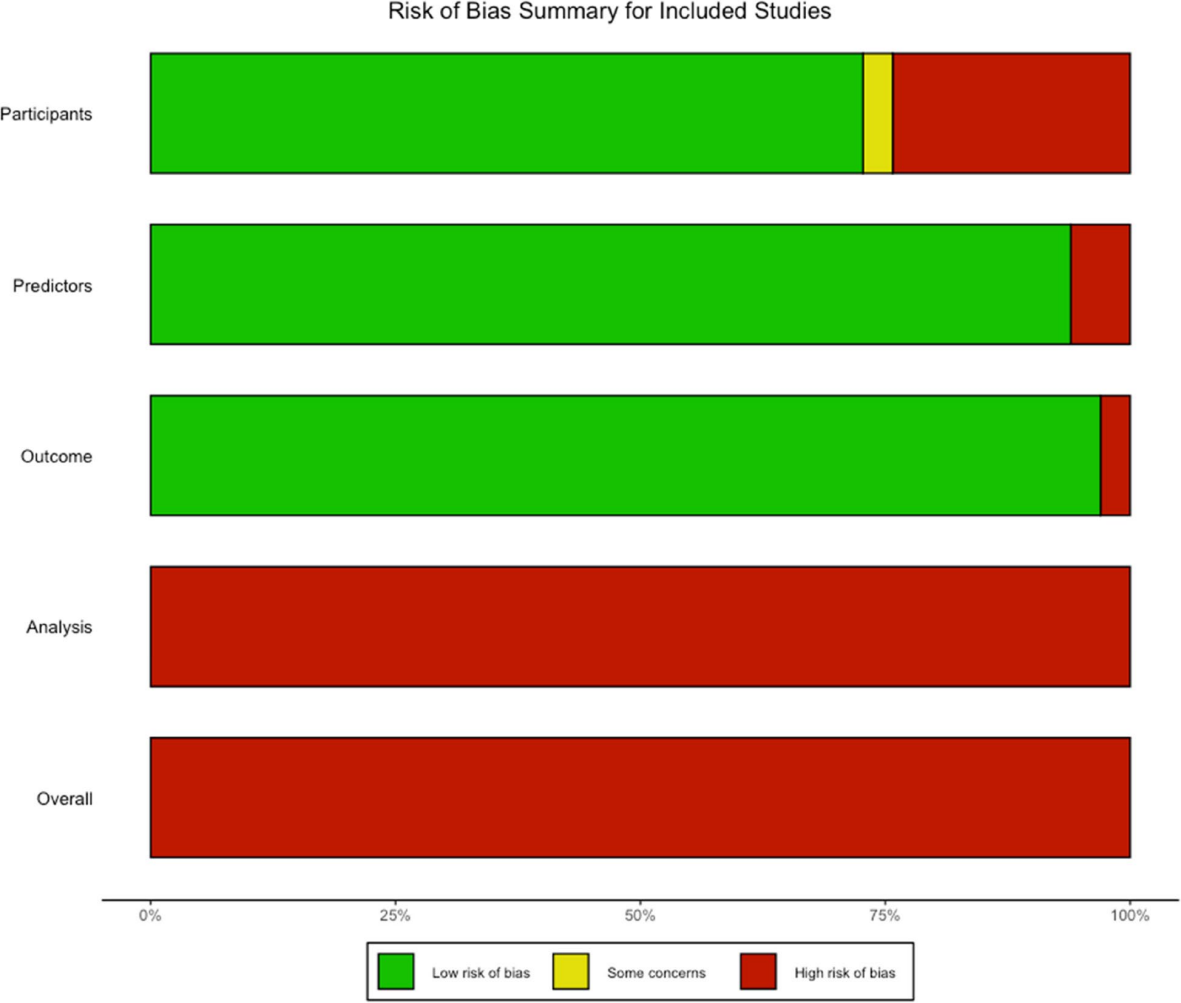
Risk of Bias Summary for Included Studies

Risk-of-bias of the studies is summarized in Fig. 7 and Fig. 8. All studies (n = 35) carried high risk of bias due to issues in analysis or its reporting. Specifically, all studies were rated as high risk in the 'Analysis' category due to inadequate reporting of calibration and mishandling of missing data. In many cases, missing data handling was either not mentioned or addressed through complete case analysis, instead of employing multiple imputation or other gold standard approaches. Eleven studies (31.4%) were considered high risk of bias in the 'Participants' domain as they used retrospective data sources rather than prospective ones. Almost all studies (n = 34) carried low risk of bias in the 'Predictors' and 'Outcome' domains, suggesting well-defined measurement of predictors and outcomes. 33 out of the 35 included studies were considered highly applicable for our study question.

## Discussion

Prior to the completion of this study, three reviews on prognostic models for patients with advanced cancer have been identified. Simmons et al., did not perform meta-analysis to compare the performances of models and the potential underlying factors [18]. Pobar et al. aimed specifically at identifying models suitable for radiation therapy planning, so only two specific timeframes (4-week and 3-month survival) were included for evaluation. [56] Owusuaa et al. included studies that involve stage 1 cancers and that study subjects were not strictly for palliative intent [57]. Our review updated the identification of prognostic models for patients with advanced cancer and presented meta-analysis to add to the literature body. Meta-analyses of specific prognostic models described in this review had been published elsewhere but whose eligibility criteria differed significantly [57]. examined PPS including non-cancer patients [58]. examined PaP involving haematological and non-terminal malignancies [59].

We have systematically searched for validated prognostic models for survival prediction among patients with advanced cancer. The identified models typically combine both clinical and objective biologic factors to estimate survival probability. Whether models solely comprised of biologic factors perform better or worse cannot be ascertained due to limited number of studies available for meta-analysis. Nevertheless, Objective Prognostic Index for Advanced Cancer (OPI-AC), an example of such models, demonstrated possible superiority of objective parameters (C-index > 0.8 for 30-day, 56-day, and 90-day survival) [60]. This represents an important research gap that may determine future directions for prognostic model development.

Our findings were largely in line with the ESMO Clinical Practice Guideline where prognostic models are endorsed for the clinical prediction of survival ranging weeks to months [60]. Further to their recommendations, we added that shorter- (days) and longer-term (months to years) survival predictions have been tested but data were relatively scarce to support clinical incorporation. The superiority of PaP over PPI in terms of discrimination, as reflected in our multi-level regression, also resemble previous cohort studies quoted in the guideline [61, 62]. While the C-index is a widely used measure of predictive accuracy, it represents only one aspect of model performance. The clinical significance of the observed difference in C-statistics between PaP and PPI remains unclear and warrants further investigation.

It is important to note that selecting a suitable model for clinical use or further validation should be guided by considerations beyond performance metrics alone. The clinical settings, prediction timeframe, and patient characteristics underlying the study sample may deviate significantly from the population of interest [63]. PaP incorporates objective laboratory factors, whereas PPI relies solely on clinical parameters. This distinction has practical implications, as PPI might be more convenient and accessible in resource-limited settings where laboratory facilities are scarce [64]. Moreover, the invasiveness of tests, the expertise required from personnel, and the complexity of assessments involved would be potential determinants of what models to be chosen in a certain clinical setting. Even if model performances do not show superiority over clinician predictions, the reproducibility and objectivity of prognostic tools may aid communication and education for less experienced staff as well as patients and their carers [65].

Hence, the choice of prognostic approach in practice may depend on a balance of factors including predictive accuracy, resource availability, ease of use, and the specific clinical context. While this study prioritized models based on their performance, a comprehensive approach considering both statistical performance and practical implementation is necessary for optimal clinical application.

Furthermore, utilizing more accurate prognostic models would theoretically facilitate end-of-life communication with patients and caregivers. However, no studies have yet been conducted to compare the impacts of different prognostication methods (clinician prediction, prognostic models, prognostic factors) in clinical care. As mentioned in the ESMO practice guideline, RCTs on the feasibility and clinical utility of various prognostication methods are warranted [66].

Several methodological limitations across included studies were identified. Cut-offs for assigning patients into prognostic groups varied between studies of the same model. For example, while [26, 66] and [25, 28] categorized those with PPI > = 6 as likely to survive less than 3 weeks, [26, 32] adopted PPI > 5 as the benchmark. Similarly, [28] treated PaP > 9 as unlikely to survive beyond 3 weeks whereas [26] adopted PaP > 10. The lack of standardization across studies obscures the evidence base of predictive performance. However, it also highlights the need to experiment with different cut-offs in a new cohort. The effect of altering cut-offs on model performance was less thoroughly studied and reported in the studies of our review.

Timing of measurement can also affect the accuracy of survival prediction. Some studies included in this review suggest that serial measurements give more reliable prediction. For instance, score changes alone and combination with initial score have been investigated for PPI [31]. Whether the same effect can be appreciated in other models remains under-explored. Regardless of this preliminary finding, timing of measurements needs to be standardized. There were appreciable variations across studies in terms of when model factors were assessed, particularly in relation to previous treatments and palliative care referral. The timing was not always clearly defined in studies either. Understandably, this is heavily dependent on resources and guidelines in localities, but alignment should be sought within one setting and that assessment time for survival prediction should be clearly defined in the journey of care. Theoretically, survival should be counted from the time assessment is done. Use of earlier or later test results for current prediction should be minimized for the purpose of model development and validation.

The body of evidence we have gathered is bound by several problems that may hinder immediate translation into clinical practice. In particular, the wide prediction intervals and high I^2^ values suggested significant heterogeneity across studies. Palliative care settings and referral criteria differ across localities [8–11]. Disease and treatment statuses of patients at and before recruitment into studies were therefore heterogeneous and altered the prognostic trajectories. However, certain studies underreport eligibility criteria and/or sample characteristics, rendering pooling and sub-group analyses difficult [20, 25, 33–35]. Moreover, dichotomization of otherwise continuous variables (such as blood results), inadequate testing or reporting of model assumptions, a lack of account for missing data, and the absence of calibration plots create uncertainties about the strength of evidence presented, as shown in the high risks of bias of many included studies [41]. With the advent of machine learning and artificial intelligence, it is essential to keep updated with newest guidelines on analysis and reporting [67]. To enable a more precise and directive recommendation from reviews and meta-analyses, future studies may compare performance of different prognostic tools in the same specific patient subgroups or healthcare contexts, such that the discrepancies in performance statistics can be pooled and whose consistencies can be assessed.

## Conclusion

Reliable prognostication is essential to inform both patients and clinicians in their planning of palliative care. This review addresses several gaps in existing literature by focusing specifically on patients with advanced solid tumors receiving palliative care, excluding hematological malignancies which have distinct disease trajectories. Through conducting comprehensive meta-analyses of model performance and providing direct comparisons between prognostic tools, our review offers insights specific to this important patient population.

Our study provided preliminary evidence that PaP had a higher discriminative ability than PPI. Yet, definitive conclusions cannot be made as many studies have significant methodological limitations such as the lack of comprehensive statistical testing, failure to report missing data handling, and omission of critical demographic information such as treatment status.

It remains uncertain if accurate prognostication methods would translate into superior clinical care. Future RCTs should investigate the clinical impacts of utilizing different prognostic models on palliative care, advanced care planning, resource allocation, hospice referrals, end-of-life discussions, etc. The feasibility, cost-effectiveness, and patient acceptance of prognostic models should be explored as well.

## Supplementary Information

Supplementary Material 1.

## Data Availability

No datasets were generated or analysed during the current study.
