# Supplementary material for: Prognostic models for survival predictions in advanced cancer patients: a systematic review and meta-analysis
Source: BMC Palliat Care. 2025 Mar 1;24:54. doi: 10.1186/s12904-025-01696-4 (PMC11871741; doi:10.1186/s12904-025-01696-4)
Supplement: Supplementary file 1 — Supplementary Material 1. [file 12904_2025_1696_MOESM1_ESM.docx]

Item 1: Search Terms

Example for OVID Medline

[1] (scor* or tool* or scale* or model*).tw.

[2] (predict* or prognostic* or estimat*).tw.

[3] (advanced or metasta* or terminal* or end-stage or incurable or end-of-life).tw.

[4] (neoplas* or cancer* or malignan* or oncolog* or carcinoma).tw.

[5] (surviv* or mortality or life expectancy).tw.

[6] (palliat* or hospice or terminal care or end-of-life care).mp.

[1] AND [2] AND [3] AND [4] AND [5] AND [6]

Item 2: Complete List of Prognostic Factors in Included Prognostic Models

| Models | Objective Factors | | Clinical Factors | |
| --- | --- | --- | --- | --- |
|  | Continuous | Categorical | Continuous | Categorical |
|  |  |  |  |  |
| Palliative Prognostic Index (PPI) |  |  |  | PPS  Oral Intake  Edema  Dyspnea  Delirium |
| Combination of initial palliative prognostic Index (PPI) and week 1 PPI |  |  |  | Initial PPI  ΔPPI at week 1 |
| PPI on discharge / PPI on admission for patients with acute concomitant disease |  |  |  | PPI on discharge / on admission |
| Survival Prediction Score (SPS): 3-variable model |  |  |  | Primary Ca site  Metastasis site  KPS |
| Number of risk factors (NRF): 3-variable model |  |  |  | Primary Ca site  Metastasis site  KPS |
| A proposed prognostic 7-day survival formula | Respiratory rate  Blood Urea Nitrogen |  |  | Cognitive status  Edema  ECOG |
| Recrusive partitioning: 2-variable model |  |  |  | KPS  Metastasis site |
| Survival Prediction Score (SPS): 6-variable model |  |  |  | Primary Ca site  Metastasis site  KPS  ESAS fatigue score  ESAS appetite score  ESAS shortness of breath score |
| Number of risk factors (NRF): 6-variable model |  |  |  | Primary Ca site  Metastasis site  KPS  ESAS fatigue score  ESAS appetite score  ESAS shortness of breath score |
| Palliative Prognostic Score (PaP) | Leukocytes count  Lymphocyte % |  |  | Dyspnea  Anorexia  KPS  Clinical prediction |
| Modified Palliative Prognostic Score - Delirium (D-PaP) | Leukocytes count  Lymphocyte % |  |  | Delirium  Dyspnea  Anorexia  KPS  Clinical prediction |
| Palliative Prognostic Score - Nomogram (PaP-Nomogram) | Leukocytes count  Lymphocyte % |  |  | Dyspnea  Anorexia  KPS  Clinical prediction |
| Cochin Risk Index Score (CRIS) |  | Gender  Urea  Transthyretin  Leukocytes count |  | KPS |
| Palliative Performance Scale (PPS) |  |  |  | Ambulation  Activity level & evidence of disease  Self-care  Oral intake  Consciousness level |
| Prognostic Scale for terminal hospitalized chinese cancer patients (8-variable) |  |  |  | KPS  Cognitive impairment  Oral intake  Dyspnea  Edema |
| A graphic tool to estimate individualized survival curves (5-variable) |  |  |  |  |
| PRONOPALL score (4-variables) |  |  |  | ECOG  Number of metastatic sites |
| Objective Prognostic Score (OPS) |  | Leukocytosis  Serum bilirubin  Serum creatinine  Serum LDH |  | ECOG  Dyspnea  Oral intake |
| Imminent Mortality Predictor for Advanced Cancer (IMPAC) | Temperature  Heart rate  Cardiac rhythm  Systolic blood pressure  Diastolic blood pressure  Pulse oximetry  Respiratory rate  White cell count  Haematocrit  Sodium  Potassium  Chloride  Blood Urea Nitrogen  Creatinine | Age | Respiratory assessment  Cardiac assessment  Gastrointestinal assessment  Nutrition/dietary assessment  Genitourinary assessment  Musculoskeletal assessment  Neurologic assessment  Peripheral vascular assessment  Braden score  Skin assessment  Psychosocial assessment  Safety assessment | AED admission  Prior hospitalization within 90 days  Primary Ca sites |
| Objective Prognostic Index for advanced cancer (OPI-AC) (7-days) | Heart rate  Urea  Albumin |  |  |  |
| Objective Prognostic Index for advanced cancer (OPI-AC) (14-days) | Heart rate  Urea  Albumin  Respiratory rate  Creatinine  CRP |  |  |  |
| Objective Prognostic Index for advanced cancer (OPI-AC) (30-days) | Heart rate  Urea  Albumin  Lymphocyte %  Total bilirubin  Platelet/lymphocyte ratio |  |  |  |
| Prognosis in Palliative Care study (PiPS-B14/56) |  |  |  | Anorexia  Dyspnea  Dysphagia  Fatigue  Weight loss |
| Six adaptable prognosis prediction model (SAP) | Albumin  LDH  Neutrophil |  |  |  |
| Nomogram based parameters to predict 90-days survival |  | Sex  Age at diagnosis  LDH  Cystatin C  Neutrophil  Haemoglobin  Uric acid  Albumin |  | KPS  TNM |
| Artifical Neural network for 30-days survival prediction | Leukocytes  Neutrophils  MCHC  CRP  Albumin |  | ESAS  KPS | PPS  Brain metastasis  Liver metastasis  Distant metastasis |
| Logistic regression for 30-days survival | Leukocytes  Neutrophils  MCHC  CRP  Albumin |  | ESAS  KPS | PPS  Brain metastasis  Liver metastasis  Distant metastasis |
| Prognostic model for advanced cancer (PRO-MAC) | White cell count  Albumin |  |  | Number of organs metastasized  PPS V.2  ESAS revised |
| Modified Barretos Prognostic Nomogram (BPN) - with laboratory values | White cell count  Albumin | Gender |  | Presence of locoregional disease  Sites of metastasis  ECOG-PS |
| Modified Barretos Prognostic Nomogram (BPN) - without laboratory values |  | Gender |  | Presence of locoregional disease  Sites of metastasis  ECOG-PS  Antineoplastic treatment |
| Machine learning (Gradient-boosted trees binary classifier) | * | * | * | * |
| Objective Palliative Prognostic Score |  | Heart rate  White cell count  Platelet  Creatinine  Potassium |  | Absence of chemotherapy |
| Clinical Model |  | Age |  | Surprise question  Cancer type  Visceral metastases  Brain metastasis  ECOG  Weight loss  Pain score  Dyspnea |
| Extended Model |  | Age  Haemoglobin  CRP  albumin |  | Surprise question  Cancer type  Visceral metastases  Brain metastasis  ECOG  Weight loss  Pain score  Dyspnea |
| Rothman Index |  | * |  | * |
| Supportive and Palliative Care Indicators Tool |  | * |  | * |
| Data mining techniques (random forest algorithms, support-vector machine algorithms, back-propagation neural network algorithms) | * | * | * | * |

*The authors did not report the full list of prognostic factors

Notes:

- Blank fields indicate that these variables were not utilized in the models

Abbreviations

KPS: Karnofsky Performance Status

ECOG: Eastern Cooperative Oncology Group (ECOG) Performance Status

ESAS: Edmonton Symptom Assessment System

AED: Accident & Emergency Department

TNM: Tumor, Node, Metastasis

PPS: Palliative Performance Scale
